# Silsesquioxane polymer as a potential scaffold for laryngeal reconstruction

**DOI:** 10.1016/j.msec.2018.07.003

**Published:** 2018-11-01

**Authors:** Nazia Mehrban, James Bowen, Angela Tait, Arnold Darbyshire, Alex K. Virasami, Mark W. Lowdell, Martin A. Birchall

**Affiliations:** a Division of Surgery, University College London, London, WC1E 6BT, United Kingdom; b School of Engineering and Innovation, The Open University, Milton Keynes, MK7 6AA, United Kingdom; c Department of Biochemical Engineering, University College London, London, WC1E 6BT, United Kingdom; d Department of Histopathology, University College London, London, WC1N 3JH, United Kingdom; e Department of Haematology, University College London, London, NW3 2QG, United Kingdom; f UCL Ear Institute, University College London, London, WC1X 8DA, United Kingdom

**Keywords:** Polymer, Larynx, Epithelial cells, Tissue engineering, Cell, Characterisation

## Abstract

Cancer, disease and trauma to the larynx and their treatment can lead to permanent loss of structures critical to voice, breathing and swallowing. Engineered partial or total laryngeal replacements would need to match the ambitious specifications of replicating functionality, outer biocompatibility, and permissiveness for an inner mucosal lining. Here we present porous polyhedral oligomeric silsesquioxane-poly(carbonate urea) urethane (POSS-PCUU) as a potential scaffold for engineering laryngeal tissue. Specifically, we employ a precipitation and porogen leaching technique for manufacturing the polymer. The polymer is chemically consistent across all sample types and produces a foam-like scaffold with two distinct topographies and an internal structure composed of nano- and micro-pores. While the highly porous internal structure of the scaffold contributes to the complex tensile behaviour of the polymer, the surface of the scaffold remains largely non-porous. The low number of pores minimise access for cells, although primary fibroblasts and epithelial cells do attach and proliferate on the polymer surface. Our data show that with a change in manufacturing protocol to produce porous polymer surfaces, POSS-PCUU may be a potential candidate for overcoming some of the limitations associated with laryngeal reconstruction and regeneration.

## Highlights

• A porous polymer was created as a scaffold for laryngeal reconstruction.
• Porous POSS-PCUU exhibited good tensile properties but limited surface porosity.
• Primary fibroblasts and epithelial cells attach to POSS-PCUU.
• POSS-PCUU may be used for larynx reconstruction by optimising manufacturing conditions.

## Graphical abstract

The manufacture and characterisation of porous silsesquioxane scaffolds for laryngeal reconstruction.

**Figure:**
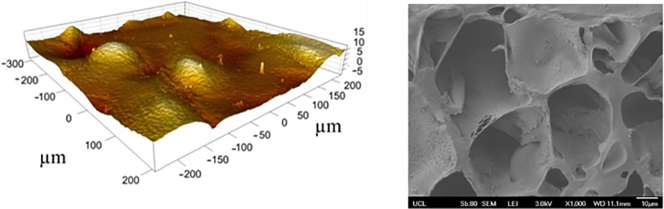

## 1 Introduction

Laryngeal cancer, disease, trauma and their treatment affect the breathing, swallowing and voice [1, 2]. Depending on the extent of the damage, the issues may be corrected by medialization, [[3], [4], [5]] laryngotracheal reconstruction [6], partial or total laryngectomy [7], or the problem may simply be bypassed by tracheostomy. In many, the damaged laryngeal tissue and its repair severely reduce the patient's quality of life [8, 9].

The human larynx sits at the crossroads of three critical functions: eating, breathing and talking. It has six linked cartilages, a finely controlled array of small muscles and delicate neurovascular supply [10]. Microscopically, the larynx has ciliated [11], goblet [12], brush [13], small granule [14] and basal [15] cells. Damage to any part of this complex structure can affect more than one critical function so its repair is not a simple task, often necessitating choosing one activity to preserve at the expense of the others. To rebuild the normal balanced complexity each element must be recreated: a formidable challenge.

Tissue engineering combines fundamental engineering theory with biological systems to create morphological, chemical and functional mimics of healthy tissue that allow better integration between the native and synthetic tissues [16, 17]. This approach hypothetically reduces the chances of the adverse immune responses associated with allotransplantation for example. One of the most common forms of tissue engineering involves introducing the patient's own cells onto a supportive temporary scaffold, providing them with sufficient nutrients to expand and differentiate before implantation at the injured/diseased site [18]. Selecting a scaffold material which will be conducive to cell growth requires mechanical strength, degradation behaviour and integration with surrounding tissue post-implantation [19, 20]. Encouraging seeded or infiltrating cells to attach, migrate, proliferate and differentiate is also challenging. Cell behaviour is guided by physical and chemical cues [21] and growth factors to interact with cell receptors [22, 23]. Scaffold material can lever any or all of these in a controlled way.

Suitable materials can be found from natural and synthetic sources [24, 25] and include, but are not limited to, polyethylene terephthalate, polytetrafluoroethylene, polyglycolic acid [26], collagen [27], gelatin [28], alginate [29], peptide-based materials [30, 31] and decellularised scaffolds [32]. However, successful implantation of these materials is still hindered by a chemical and physical mismatch between the native and engineered tissues [33]. Often materials that are mechanically stable do not encourage cell growth while materials that do encourage cell proliferation and differentiation are usually mechanically weak [34]. For this reason a combinatorial approach, bringing together two or more materials to create novel hybrid scaffolds has been proposed [35, 36]. These hybrids may be fully synthetic [37], natural [38, 39] or a mixture of the two [36].

Polyhedral oligomeric silsesquioxanes (POSS) are a candidate class of materials with cage-like structures composed of silicone and oxygen. The external surface of the POSS nanocage is composed of easily modifiable organic moieties, usually hydrocarbons [40]. Many researchers have modified these groups to create hybrid materials for a range of purposes, such as thermal and mechanical stability for circuit printing [41], thermosetting polymers [42], to remove sulphur from fuels [43] and for sea water desalination [44]. Modification of POSS-nanocage exterior has also allowed medically-relevant materials to be developed [[45], [46], [47], [48]]. POSS-PCUU is a hybrid of POSS and poly(carbonate urea) urethane, a member of the widely-used polyurethane polymer family [[49], [50], [51]].

POSS-PCUU has been shown to have suitable mechanical properties to retain the formed geometry in vivo [[52], [53], [54]]. Here, we use a precipitation and porogen-leaching method to create porous scaffolds and assess the effect of increasing concentrations of porogen on material properties including chemistry, surface morphology, roughness and wettability, internal porosity, mechanical integrity and cell compatibility. While previous studies have explored the use of POSS-PCUU in vitro and in vivo, to our knowledge, this is the most comprehensive, analytical study conducted on porous POSS-PCUU to date.

## 2 Materials and methods

### 2.1 POSS-PCUU manufacture and scaffold formation

#### 2.1.1 POSS-PCUU manufacture

All chemicals for polyhedral oligomeric silsesquioxane poly(carbonate-urea) urethane (POSS-PCUU) manufacture were purchased from Sigma Aldrich Ltd. (UK) unless stated otherwise. Briefly a polyol blend was formed by heating a mixture of *trans*-cyclohexanechloroydrinisobutyl-silsesquioxane (Hybrid Plastics, USA) and polycarbonate polyol (2000 MW) to 130 °C. After cooling the solution to 80 °C a prepolymer solution was formed by adding flake 4,40 - methylene bis(phenyl isocyanate) and heating the mixture at 70–80 °C for 120 min. To the prepolymer solution, dimethylacetamide (DMAC) was added dropwise and cooled to 35 °C. A mixture of ethylenediamine in DMAC was then added dropwise to extend the polymer chain and form POSS-PCUU. The resulting solution was stored at room temperature until use.

#### 2.1.2 Particle size distribution and sphericity

Particle size distributions were measured using a QICPIC powder size analyzer, operated using VIBRI and GRADIS units (Sympatec, UK). Videos were analysed frame-by-frame to yield density distributions (q3) from which the following statistical parameters were calculated according to well established procedures [[55], [56], [57]]: x_10_, x_50_, x_90_, Sauter Mean Diameter (SMD), Volume Mean Diameter (VMD), aspect ratio, and sphericity.

#### 2.1.3 POSS-PCUU scaffold formation

Scaffold solutions were created by mixing POSS-PCUU with sodium hydrogen carbonate (NaHCO_3_) particles sieved at 25-53 μm (or 53-100 μm where stated; Fig. S1). Tween 20 was also added at 2% (w/w). The ratio of NaHCO_3_ to POSS-PCUU for each sample is shown in Table 1.

**Table 1:** Quantity of NaHCO_3_ in POSS-PCUU and DMAC for each sample type.

Sample	NaHCO_3_ in POSS-PCUU + DMAC (% w/w)	NaHCO_3_ in POSS-PCUU (% w/w)
1	0	0
2	1	5.7
3	5	24
4	10	40.1
5	20	60.1
6	30	72.2
7	40	80.2
8	50	86
9	50 (53-100 μm)	86

**Fig. 1:**
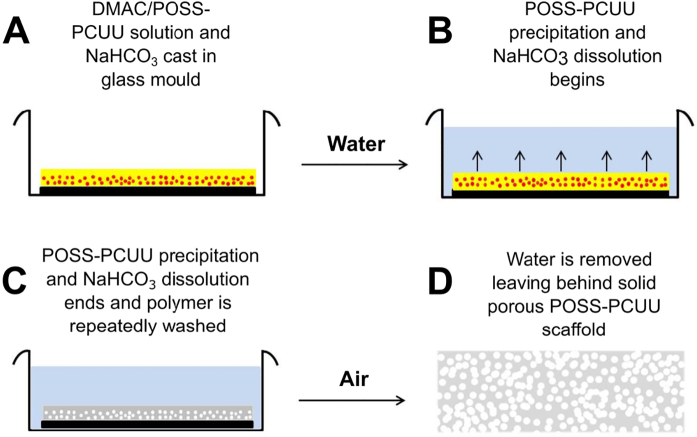
Manufacture of POSS-PCUU. (A) DMAC/POSS-PCUU solution (yellow) and NaHCO_3_ (red) cast in glass mould (black) in air (white). (B) Water (blue) added to mould to begin solvent exchange and promote POSS-PCUU precipitation and NaHCO_3_ dissolution. (C) POSS-PCUU precipitation and NaHCO_3_ dissolution ends and the polymer (grey) is repeatedly washed with water. (D) Water is removed leaving a solid porous POSS-PCUU scaffold. (For interpretation of the references to colour in this figure legend, the reader is referred to the web version of this article.)

The solution was mixed using a centrifugal mixer (2000 rpm, Thinky ARE-250, USA) and any air bubbles removed through a ‘degassing’ cycle (1500 rpm) on the mixer. The mixed solution was then poured onto a clean glass mould measuring 148 × 210 mm which was surrounded by a ~300 μm thick autoclave tape perimeter. To precipitate the polymer and allow NaHCO_3_ particles to leach out of the scaffold the glass mould was slowly submerged into a bath containing 5 L of deionised water (DI H_2_O; Fig. 1). The DI H_2_O was replaced 3 times a day for 5 days before the precipitated porous polymer was peeled off the glass mould and stored in 70% ethanol (v/v ethanol in water). For polymer characterisation studies the scaffold was first washed overnight in DI H_2_O while for cellular studies the samples were autoclaved at 121 °C for 20 mins in DI H_2_O before use (Fig. S2). For the purpose of this study we refer to precipitated polymer as 'scaffold'.

### 2.2 Material characterisation

#### 2.2.1 Raman spectroscopy

The surface chemical composition of the precipitated POSS-PCUU polymers was evaluated using a confocal Raman microscope (WiTec Alpha 300R, LOT Oriel, UK) with a 0.3 W single frequency 785 nm diode laser (Toptica Photonics, Germany). Data were collected via an Acton SP2300 triple grating monochromator/spectrograph (Princeton Instruments, USA) over a 200–3000 cm^−1^ wavenumber range at a mean spectral resolution of 3 cm^−1^.

#### 2.2.2 X-ray photoelectron spectroscopy

The composition and chemical bonds of the polymer surface were investigated using X-ray photoelectron spectroscopy (XPS). Analysis was performed using a K-alpha X-ray Photoelectron Spectrometer (Thermo Fisher Scientific, UK), operating a microfused, monochromated Al Kα X-ray source with a spot size of 400 μm and a power of 36 W. The step size in order to obtain individual peaks was 0.1 eV, whereas 1 eV was used for the acquisition of a full spectrum over the complete range of binding energies (BEs). The vacuum pressure in the analysis chamber was <10^−9^ mbar. The C 1s (BE = 285 eV), N 1s (BE = 400 eV) and O 1s (BE = 531 eV) photoelectron peaks were analysed in detail. Three non-overlapping regions were measured for each sample. Data were fitted using the Gauss-Lorentz function, and the Shirley method was used for background subtraction. CasaXPS software was used for data processing.

#### 2.2.3 Field emission scanning electron microscopy

The microstructure of precipitated POSS-PCUU polymer samples was evaluated by field emission scanning electron microscopy (FEG-SEM, Quanta 200F, FEI, USA). The samples were first dehydrated in graded ethanol 20%, 30%, 50%, 70%, 90% and 100% v/v ethanol in water for half an hour each. The ethanol was then exchanged for liquid CO_2_ and the samples were critical point dried at 1040 psi and 32 °C in a critical point dryer (K850, Quorum Technologies, UK). Dried specimens were cut using a scalpel to show surface detail as well as the cross-sections. Once mounted onto stubs the samples were sputter coated with 15 nm of gold (Au; Q150T ES, Quorum Technologies, UK). Micrographs of polymer structure were captured at an accelerating voltage of 5 kV and a working distance of 8 mm.

#### 2.2.4 Profilometry

Profilometry was employed to evaluate the topography and roughness of polymer surfaces. Measurements were performed using a DCM3D microscope (Leica Microsystems, UK), using a white light source. Samples were imaged using a 20× objective lens, which corresponded to an analysis window of dimensions 637 μm × 477 μm. Scanning Probe Image Processor software (Image Metrology, Denmark) was employed for the analysis of acquired images, yielding the average roughness, Sa, as a measure of the surface roughness. Each value presented is the mean of a minimum of five measurements at separate locations.

#### 2.2.5 Wetting behaviour

The wetting behaviour of polymer surfaces was assessed using a DSA 100 Drop Shape Analyzer (Krüss, UK), employing DI H_2_O as the analyte. Samples were critical point dried prior to analysis, as outlined earlier. Droplets of volume 5 μL were deposited onto the surface through a flat-ended needle. All data were collected at temperatures in the range 20–22 °C and relative humidity in the range 40–60%.

#### 2.2.6 Helium pycnometry

The true density of POSS-PCUU specimens were determined using helium pycnometry. Samples were first dried using the critical point drying method outlined earlier. Sample masses were then measured using a balance accurate to ±0.1 mg (Sartorius, UK). Sample volumes were measured using a Helium Pycnometer (AccuPyc II 1340, Micromeritics, UK), employing 20 measurements per sample; the sample chamber was allowed to equilibrate at 134 kPa for each measurement cycle. Hence, the true density could be calculated.

#### 2.2.7 Mercury porosimetry

Samples were dehydrated and critical point dried as outlined earlier. POSS-PCUU porosity and pore size distribution were measured by adding the sample to a glass penetrometer (3 mL bulb volume; 1.1 mL stem volume), evacuating air from the penetrometer and sample, then intruding mercury under controlled pressure. A pressure range of 3 kPa–207 MPa was employed (AutoPore IV, Micromeritics, UK). The Washburn equation was used to determine the pore size distribution for each sample. The bulk density of the sample was estimated using the volume excluded within the penetrometer at the lowest intrusion pressure.

#### 2.2.8 Laser cutting

For tensile tests polymer sheets were cut into dumbbells with a 20 mm gauge length and 4 mm width using a Trotec Speedy 100R laser cutter (Trotec Laser, UK) at 10 W, 0.6 V and a pulse rate of 1 kHz.

#### 2.2.9 Tensile strength

The tensile strength of dumbbell-shaped polymers (n = 6 per sample type) was measured using an Instron 5565 mechanical tester (Instron Ltd., UK) at a rate of 50 mm/min. For each dumbbell tested the thickness was separately measured and recorded.

### 2.3 Cell studies

#### 2.3.1 Cell studies

Primary porcine fibroblasts (FB) and epithelial cells (EC) were donated by Mark Lowdell, University College London. For cell culture studies on POSS-PCUU the polymer was cut into 7 mm circles using the laser cutting method outlined in Section 2.2. These were autoclaved at 121 °C for 20 min (Priorclave, BioCote, UK) and inserted into 96-well plates before seeding.

At 70%–80% confluency cells were washed with PBS and the FB feeder layer dissociated using 5 mL ethylenediaminetetraacetic acid (EDTA, 0.2%, Sigma Aldrich Ltd., UK) for 5 mins at 37 °C. The feeder layer was then removed and 6 mL TrypLE was added to digest the EC for 10 mins at 37 °C. Digested cells were diluted with equal volume modified Greens Medium (M-Greens; containing 330 mL (DMEM; Gibco, UK), 110 mL Ham's F12 Nutrient Mixture (F-12, Sigma Aldrich Ltd., UK), 50 mL foetal bovine serum (FBS; Gibco, UK), 0.4 μg/mL hydrocortisone (R&D systems Inc., USA), 10 μg/mL human recombinant epidermal growth factor (Bio-Techne Ltd., UK), 5 μg/mL insulin (Actrapid, Novo Nordisk, Denmark), 250 μg isoproterenol (Calbiochem, Merck Millipore, USA), 1% Anti:Anti, 250 μg ciprofloxacin and 25 μg gentamycin and then centrifuged at 300 g for 5 mins (Allegra X-15R, Beckman Coulter, UK)) before being resuspended in M-Greens containing 10 mM ROCK inhibitor Y-27632 (Cell Guidance Syetms, UK). FB were digested with 5 mL TrypLE alone and resuspended in Dulbecco's Modified Eagle Medium (S-DMEM) supplemented with 10% v/v FBS and 1% v/v non-essential amino acid solution (Thermo Fisher Scientific, UK). Both FB and EC were used at passage 3 and 8 for FB and passage 3 for EC. The digested cells were seeded onto the polymer scaffolds at a density of 3.78 × 10^4^ FB and 1.14 × 10^5^ EC per scaffold in M-Greens.

#### 2.3.2 Sample embedding and sectioning

For histology the samples were fixed in 10% formal saline (containing 10 mL 40% formaldehyde (Acquascience, UK), 0.9 g Sodium chloride and 90 mL distilled water) for a minimum of 7 h prior to processing. Samples were then dehydrated through an alcohol gradient (100% Industrial Methylated Spirit (IMS), 95% IMS, 70% IMS, Acquascience, UK), followed by chloroform clearance on a Leica Peloris II tissue processing machine (Leica Biosystems, Germany) and incubation in molten (>60 °C) paraffin wax (CellPath Ltd., UK) for 1 h prior to embedding using a Sakura Tissue-Tek tissue embedding machine (Sakura, Japan). Each sample disc was bisected, with one half embedded in a vertical orientation on the cut surface, and the other half embedded horizontally. Sections were cut on a Thermo Scientific HM340E manual rotary microtome (Thermo Fisher Scientific, USA) at a section thickness of 3 μm, using Feather S35 microtome blades (Feather Safety Razor Co., Japan) before being mounted on Leica Xtra Adhesive slides (Leica Biosystems, Germany) and oven dried at 70 °C for 60 min.

#### 2.3.3 Histology

Sections were heated for 60 mins at 70 °C prior to staining with haematoxylin and eosin (H&E) using a Leica XL automated stainer with a Leica CV5030 automated coverslipper (Leica Biosystems, Germany). The staining protocol used included 2 Xylene (Acquasience, UK) dewaxing steps (2 mins each), 3 Xylene clearance steps in IMS (2 mins each) followed by staining in Harris Haematoxylin (Leica Biosystems, Germany; 5 min), washing the section, immersion in 1% hydrochloric acid in 50% IMS (10 s), wash 5 min), Eosin (Leica Biosystems, Germany), 3 dehydration steps in IMS (2 mins each), 2 alcohol clearance steps in Xylene (2 mins each) before mounting in Pertex mountant (CellPath Ltd., UK) and covering with a coverslip.

### 2.4 Statistical analysis

Data are presented as “mean ± standard error of the mean”. Significant differences in the mean values between groups were determined by ANOVA (Analysis of Variance) with post-hoc Tukey-Kramer HSD (Honestly Significant Difference) test. The significance level was set at p < 0.05.

## 3 Results

### 3.1 Material chemistry and surface properties

**Fig. 2:**
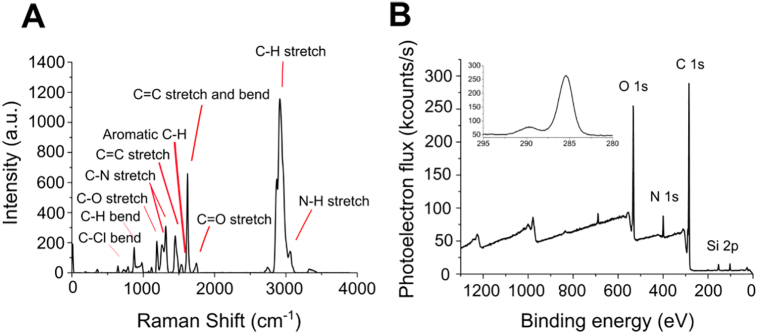
Chemical characterisation of POSS-PCUU scaffold surfaces. (A) Representative Raman spectrum of POSS-PCUU surface with bond information outlined. (B) Representative X-ray photoelectron spectrum of POSS-PCUU surface indicating presence of O 1s, N 1s, C 1s and Si 2p. Survey spectrum inset.

The formation of solid uncontaminated POSS-PCUU was confirmed by Raman spectroscopy, specifically through the presence of expected bonds in accordance with the chemical structure (Fig. 2A and S3). Furthermore, Raman spectroscopy did not indicate the presence of unreacted bonds. X-ray photoelectron spectroscopy showed that C, N, O and Si are present at the POSS-PCUU surface, once again as expected. The C 1s photoelectron spectrum clearly shows a range of binding environments comparing well with the bonds given in Fig. 2A (Fig. 2B).

**Fig. 3:**
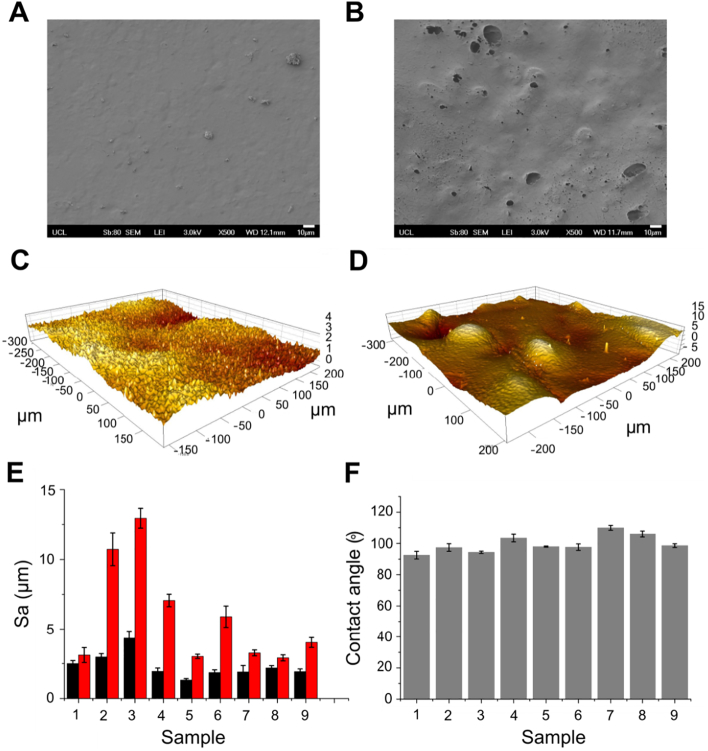
Surface morphology of POSS-PCUU scaffolds. (A) Representative scanning electron micrographs of PGI and (B) PAI surfaces. (C) Representative 3D profiles of PGI and (D) PAI surfaces with (E) average roughness of the PGI (black) and PAI (red) surfaces. (F) Water contact angle measured on the PGI surface. Scale bar for scanning electron micrographs: 10 μm. (For interpretation of the references to colour in this figure legend, the reader is referred to the web version of this article.)

While the chemistry of the material was consistent across sample types we found that the two surfaces presented by each scaffold had very different morphologies. The polymer-glass interface (PGI) surface had a smooth appearance when imaged using SEM (Fig. 3A) while the polymer-air interface (PAI) surface appeared porous and non-uniform even where open pores were not easily visible (Fig. 3B). This was confirmed through optical profilometry (Fig. 3C–D) which revealed that the PGI surface exhibited a topography with average roughness in the range 1.3–4.3 μm (Fig. 3E), while the PAI surface topography presented defined peaks (Fig. 3D) and exhibited a higher average roughness in the range 2.9–12.9 μm (Fig. 3E). While the glass interface is the likely cause of lower surface roughness for the PGI samples it was noted that the surface roughness also varied between sample types with samples 2 and 3 having the roughest PAI surface (10.7 μm and 12.9 μm) and samples 5 and 8 having the smoothest PAI surface (both 3.0 μm). The NaHCO_3_ content in these samples varies greatly from 1% and 5% w/w (for samples 2 and 3) and 20% and 50% w/w (for samples 5 and 8). No immediate trend in PAI surface roughness and sample composition is apparent. Furthermore, while there was no clear correlation between surface roughness and sample drying technique (Fig. S4) critical point drying was found to retain the planar sheet-like sample morphology without distorting the shape of the polymer. The surface morphology did not affect the overall water wetting behaviour of the material which was similar across sample types (contact angle ranging 92.5–110°, Fig. 3F). These data show that while the chemistry of the materials is not affected by changes in NaHCO_3_ content, there is a noticeable effect on polymer surface morphology, specifically at the interface with air.

### 3.2 Internal structure and porosity

**Fig. 4:**
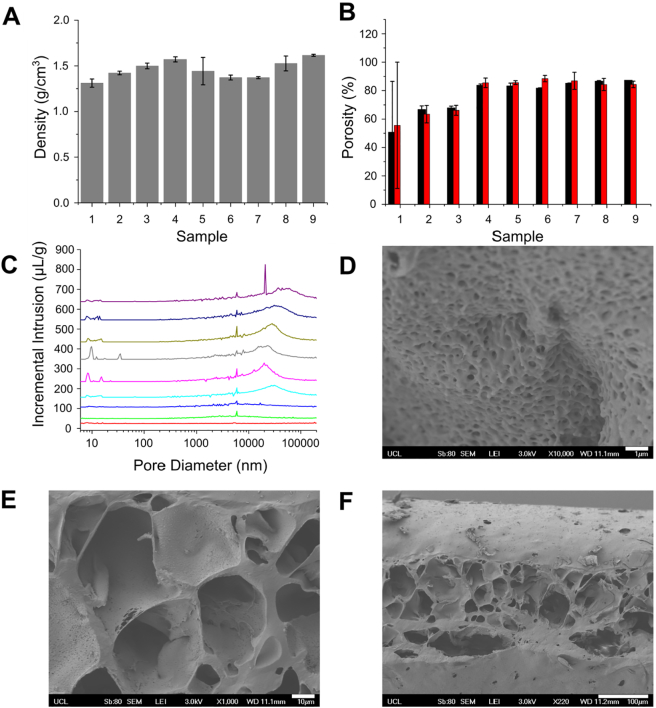
Internal porosity of POSS-PCUU scaffolds. (A) Measured density of scaffolds. (B) Porosity of scaffolds calculated (black) and measured through mercury porosimetry (red). (C) Pore diameter determined through porosimetry for Samples 1 (red), 2 (green), 3 (blue), 4 (light blue), 5 (pink), 6 (grey), 7 (dark green), 8 (dark blue) and 9 (purple). Scanning electron micrographs of polymer internal structure indicating presence of (D) nanopores and (E) micropores throughout the scaffold as evidenced by (F) the cross-sectional view. Scale bar for scanning electron micrographs: (D) 1 μm, (E) 10 μm and (F) 100 μm. (For interpretation of the references to colour in this figure legend, the reader is referred to the web version of this article.)

The true density of sample types 1–9 was in the range 1.25–1.60 g/cm^3^; this inconsistency suggests that the incorporation of closed pores is a possible outcome of the manufacture process (Fig. 4A). Samples 4–9, manufactured using >40% w/w NaHCO_3_, exhibited porosities in excess of 80% indicating an interconnected porous network. This would suggest 40% w/w NaHCO_3_ is the minimum needed to create a porous network that would allow cellular migration in the volume of polymer used to manufacture samples for this study. It was however noted that sample 1 (containing no NaHCO_3_) had the greatest variation in porosity from both calculated and measured porosities. This indicates heterogeneity in the manufacturing process. As there is no NaHCO_3_ present in these samples, these data could allude to an effect of the mixing process; possibly an inefficient ‘degassing’ stage post-mixing.

Analysis of pore size distributions revealed that as the NaHCO_3_ concentration was increased the peak pore sizes also increased from <10 μm (samples 1–3) to approx. 20–40 μm (samples 4–7) to approx. 30–50 μm (sample 8), other than where large particle size was deliberately used (sample 9); here the majority of the pores lay between 50 and 90 μm (Fig. 4C) as expected from using porogen separated using a sieve with size range 53–100 μm. Pores at 5–7 μm and smaller, <0.01 μm, were also measured giving the scaffold a fibrous appearance. Both small (Fig. 4D) and large (Fig. 4E) pores were visible via FEG-SEM which showed that although the internal structure of the polymer is highly porous there are areas where the pores appear inaccessible. Cross-sectional imaging showed that access to these from the outside of the polymer was also limited with very few pores present on the surface. Pores visible inside the polymer exhibited a variety of geometries (Figs. 4F and S5).

### 3.3 Tensile properties

**Fig. 5:**
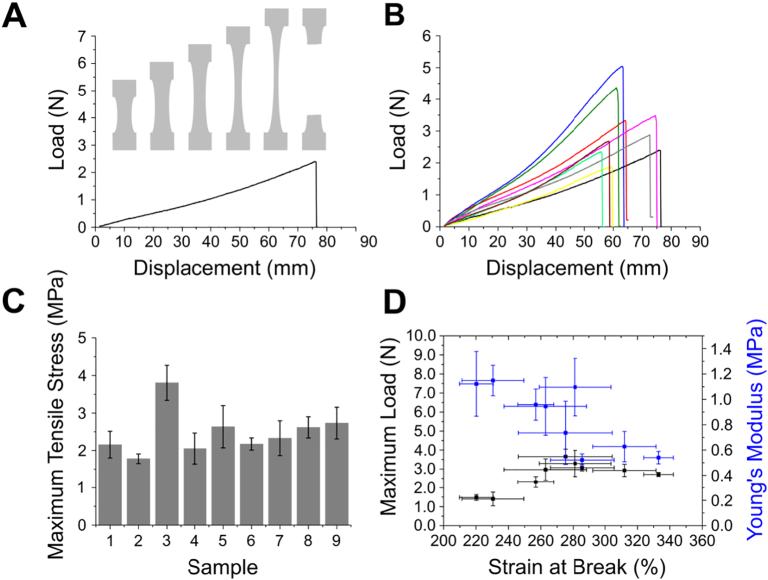
Tensile strength of POSS-PCUU scaffolds. (A) Representative maximum extension for POSS-PCUU in tensile mode with schematic showing changes in polymer cross-sectional area until the point of break. (B) Representative load-displacement for the maximum extension of sample type 1 (black), sample type 2 (red), sample type 3 (blue), sample type 4 (pink), sample type 5 (green), sample type 6 (grey), sample type 7 (yellow), sample type 8 (light green) and sample type 9 (brown) and (C) the maximum tensile stress recorded for each sample type. (D) Relationship between the maximum load, Young's Modulus and the strain recorded at the point of break for all POSS-PCUU samples. (For interpretation of the references to colour in this figure legend, the reader is referred to the web version of this article.)

The elastic behaviour of the polymer was significantly affected by the decrease in cross-sectional area during measurement of tensile strength. As displacement increased the cross-sectional area decreased due to deformation of the pore structure (Fig. 5A). Although some variability in the maximum extension was observed between sample types in the displacement range 55–76 mm, corresponding to 175–280% strain, this behaviour was exhibited by all samples (Fig. 5B). A comparison of the maximum tensile stress calculated for the samples, based on the deformed cross-sectional area, showed that while the mean stress was approximately 2 MPa for sample types 1–2 and 4–9) that recorded for sample type 3 was almost double (a mean of 3.8 MPa; Fig. 5C). A slight correlation was observed between the maximum load and strain at break for all sample types (Fig. 5D) but any correlation between the Young's modulus and the strain at break was skewed by the cross-sectional area decrease during tensile testing.

### 3.4 Cell migration and attachment

**Fig. 6:**
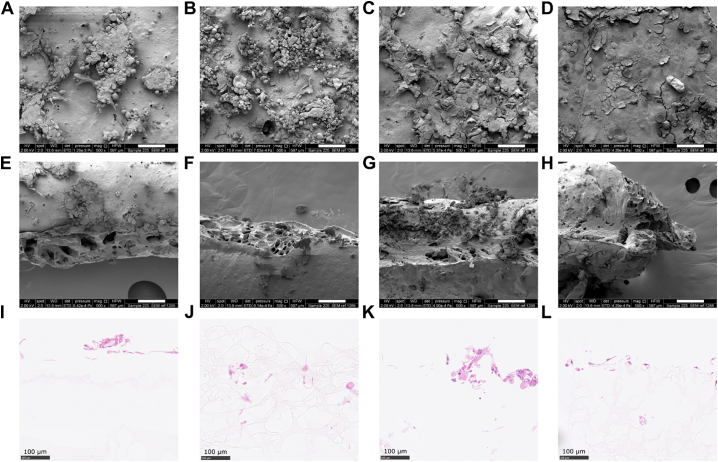
Cell attachment and migration through POSS-PCUU scaffolds after 3 days in culture. Representative electron micrographs showing cell attachment on the surface of polymer type (A) 1, (B) 5, (C) 8 and (D) 9. Cell migration through POSS-PCUU scaffolds represented by cross-sectional electron micrographs; (E) 1, (F) 5, (G) 8 and (H) 9, haematoxylin-stained (blue-purple) and eosin-stained (pink) cell images; (I) 1, (J) 5, (K) 8 and (L) 9. Scale bar: 100 μm. (For interpretation of the references to colour in this figure legend, the reader is referred to the web version of this article.)

Although cells did not attach well to the surface of the polymers on day 1 (Fig. S6), by day three it was evident that as the NaHCO_3_ concentration was increased cell attachment and proliferation also increased (Fig. 6A–C); particularly where a larger NaHCO_3_ size distribution was used (Fig. 6D). Cell distribution across the surface of all samples showed that the cells mainly expanded in clusters (Fig. 6A–B) with these clusters getting closer together as the NaHCO_3_ concentration increased (Fig. 6C) until individual cells were no longer visible (Fig. 6D). Both FB and EC were visible across all surfaces with spindle-shaped morphology (typical of FB) and cobble-stone-like morphology observed (indicating presence of EC). Capturing cross-sectional electron microscopy images of cells migrating into the scaffold was difficult due to the highly deformable nature of the polymer which meant that positioning on the sample stage was difficult (Fig. 6E–H). Histological sections, due to processing technique, were more successful and revealed that although cells did appear to adhere to the polymer, including polymers that contained no NaHCO_3_ (Fig. 6I), the majority of the cells remained on the surface of the polymer (Fig. 6I and L) with little migration into the scaffold visible (Fig. 6J and K).

## 4 Discussion

We explored whether POSS-PCUU possesses some of the characteristics ideally required for the demanding internal and external specifications of a laryngeal structural replacement. We demonstrated that porous POSS-PCUU can be formed by introducing NaHCO_3_ to the polymer solution before precipitation and that the resulting scaffold contains a range of topographical features depending upon whether the interface is with air or the glass in which it was moulded. This causes a change in the material's surface morphology and roughness. Meanwhile, the internal porous structure affects the scaffold's behaviour during tensile extension. Both of these properties are associated with changes in fibroblast and epithelial cell attachment and proliferation on the scaffold.

The formation of POSS-PCUU scaffold is a two-part process: the mixing and exchange of DMAC with water causing the polymer to precipitate out of solution and the dissolution of NaHCO_3_ to form a sponge-like scaffold through the well-established particulate leaching method [58]. As with other systems that use the same method of manufacture, sometimes referred to as ‘salt leaching’ or ‘solvent casting’, precipitation begins as the polymer solution is lowered into the water bath [59], and so we posit that a polymer membrane is rapidly formed via DMAC-water exchange and precipitation on the surface of the polymer solution creating a ‘membrane’ [60, 61], while NaHCO_3_ dissolution within the polymer is a much slower, diffusion-governed process [62]. We further suggest that the pores formed immediately below this membrane may be created by hydrodynamic instability at the interface between the polymer and the water which either through the Gibbs-Marangoni effect [63], turbulence at the interface caused by the immersion of polymer into excess water or a combination of the two, causes defects to form at the surface. These defects may manifest as finger-like pores or macrovoids, which extend into the polymer as it precipitates [60, 64]. Such columnar macrovoids have previously been noted in other polymeric scaffolds including poly(vinyliden fluoride) [65], polyether sulfone [66] and polyethylene glycol doped poly(vinyliden fluoride)-*co*-hexafluoropropylene [67]. While we observe these macrovoids at the PAI, particularly in polymers containing no NaHCO_3_, we also note that they do not occur on the PGI in our studies. We postulate that the glass mould used in our manufacturing method presents a barrier, such that water molecules do not approach the PGI from below, preventing the formation of pores via this process. We also note that ‘protuberances’ are visible below the PAI membrane. These protuberances may be caused by a rapid thickening of the polymer surface, creating a membrane [68] above the pores, through the exchange of solvent at the interface between the polymer solution and water, and therefore the formation of two surfaces with different topographical features, as noted. Meanwhile at the PAI as polymer precipitation continues it is likely that the macrovoids (i) expand into the polymer to a certain depth beyond which the energy required to form the voids begins to dissipate (ii) neighbouring voids merge to create significantly larger pores and therefore a range of pore sizes, or (iii) pores form through the dissolution of NaHCO_3_ under the membrane. A similar effect in a study by Wongchitphimon et al. [67] was attributed to phase demixing, an effect which was almost eliminated by raising the water temperature to 40 °C and promoting rapid phase demixing.

During our studies we also noted a plateau at around 86% porosity which we attribute to limitations in the inherent packing properties of the NaHCO_3_ particles [69, 70]. Porosity in scaffolds is an essential feature for guiding cell migration and creating a three-dimensional tissue for laryngeal applications [71]. Here we note that there is a clear shift in the pore size distribution with the size of the pores increasing as the porogen concentration is increased. Furthermore, as the total volume of the solutions remained consistent and only the NaHCO_3_ concentration was increased for each polymer during the manufacturing process, the pores formed from porogen leaching are likely to occupy closer spaces until some merge to form larger pores. Whilst the pore volume is expected to correspond approximately to porogen volume, this was not always the case as imaging of the internal structure shows a heterogeneity in pore size. We posit that some of the particles, as the porogen concentration is increased, begin to adhere to one another before the dissolution process is complete. This is evidenced by our analysis of particle size which showed that there is significant overlap in particle diameter between the two distributions of NaHCO_3_ particles used in the study; further supported by the increasingly aspherical morphology of the larger particles in the distribution. We also note that the high pressures used for mercury porosimetry, on the order 200 MPa, can lead to rupturing of thin membranes [72] and that there may be isolated pores within the system that are not measured at all; these pores are surrounded by polymer which cannot be reached by the mercury and are therefore not included in the pore size distribution. Isolated cavities such as these is a recognised issue with porogen leaching methods and this has partly led to gas foaming methods becoming more popular in the production of porous polymeric scaffolds [73]. It is possible that by incorporating a gas foaming technique in our system we would also observe higher interconnectivity between pores.

While pore sizes on a similar length-scale to the NaHCO_3_ particle size incorporated into the POSS-PCUU solution were expected, our investigations also revealed the formation of smaller nanopores, even in the absence of porogen. It is suggested that these pores form from the DMAC leaving the system slowly via mixing and exchange with water, as the polymer precipitates [74, 75] leaving behind nanovoids. While these pores are too small to allow cell migration, they did create a fibre-like morphology which could encourage cell attachment and scaffold integration into the body by mimicking the structural complexity of natural tissues and proteins [76], such as fibrous elastin and collagen which run vertically along the length of the vocal folds and contribute to tissue vibration and phonation [77, 78].

The presence of micro- and nanopores resulted in foam-like scaffold interiors. Some large micropores were also seen on the surface of the polymer although the concentration of these remained low. Where these were absent cells were prevented access to the internal scaffold structure. Cells that were able to migrate through the surface pores were further inhibited by reduced interconnectivity in some areas of the scaffold. This is reflected in the cellular imaging in this study where cells appear to attach and proliferate on the surface with very little migration into the scaffold. Although the obvious assumption is that the isolated pores are created by the concentration of NaHCO_3_ within the scaffold being too low and the porogen particles being dispersed across the scaffold rather than coming into contact with each other, this is unlikely to be the case as even at high porosity very few cells are seen within the scaffold. We again suggest that the polymer precipitates immediately upon contact with water while NaHCO_3_ dissolution is a slower process leading to the formation of isolated pores. Although these observations were not investigated further for the current work, it remains an important variable to investigate for future studies, particularly as an ideal tissue engineering scaffold should promote the development of a three-dimensional tissue [79]; which includes the proliferation of cells within the scaffold [80].

Furthermore, all of the measured characteristics; the shape and dimensions of the pores, the presence or absence of interconnectivity, and the density and thickness of the polymer, will all contribute towards the mechanical behaviour of the polymer [81]. Native larynges undergo a range of mechanical changes from the Bernoulli effect of airflow [82, 83] to extension and recoil in the vocal folds during swallowing and coughing for example [84, 85]. POSS-PCUU exhibits high tensile strength but as the polymer is stretched the deformation and elongation of pores causes a change in stress distribution across the scaffold. In regions which experience high stress concentrations, for example at junctions between neighbouring pores, fracture points are created that then propagate. The polymer undergoes gross deformation which contributes to the failure of the scaffold as the stresses continue to increase across an ever smaller cross-sectional area. Adding a second material to the POSS-PCUU mixture could reinforce the polymer, as evidenced by Kim et al. with the addition of fibrillar collagen in their hyaluronic acid study [86]. However, from our studies we know that the pores are geometrically anisotropic which, due to a reduction in cross-sectional area during elongation, adds to the complex behaviour observed. Therefore, a difference in the point of failure between sample types is expected. We also note that the thickness of the sample makes a difference to the polymer's mechanical strength. Whilst we had insufficient data to test this hypothesis fully, the relationship between sample thickness and Young's modulus may provide answers to the variability observed in tensile behaviour.

The chemistry of the precipitated polymer was also investigated. There was no change in chemistry between sample types and the water contact angle behaviour of the polymer surface was in-keeping with expectations for hydrophobic materials of this type [87]. The polymer presents a hydrocarbon-rich surface which does not exhibit deprotonatable moieties. Although hydrophilic surfaces reportedly are more supportive of mammalian cell growth, we show that cells remain viable on our hydrophobic scaffold surface too, with typical FB and EC morphologies observed [88]. We also noted that the density of the cells on the polymer surface increased between days 0 and 3. This suggests that the chemistry of the precipitated polymer surface did not present significant issues to cell compatibility. However, to truly create an implantable scaffold, which integrates with surrounding healthy tissue, the attachment and differentiation of the cells, with the development of mature vascular and ECM matrices, requires investigation [79].

If combined with increased porosity and better interconnectivity between the pores this scaffold could serve as a suitable candidate for tissue engineering. Although increasing porogen content is likely to increase the viscosity of the polymer solution, making it difficult to handle in the current manufacturing technique an alternative method of manufacturing, such as using extrusion to deposit highly viscous solutions, which would also assist with particle alignment and spacing, could result in a more homogenous pore structure within the scaffold, and present a way of overcoming these issues.

## 5 Conclusion

We present a porogen-leaching technique for manufacturing porous POSS-PCUU using NaHCO_3_. The porogen was easily dissolved creating a foam-like scaffold. The scaffold exhibited a non-linear relationship between load and extension under tensile deformation. Pores present within the scaffolds varied in their size distribution which contributed to this behaviour.

The scaffold chemistry remained consistent and supported the proliferation of fibroblasts and epithelial cells over three days, although the formation of a skin on the surface of the polymer prevented the migration of cells into the scaffold. Furthermore, we observed differences in topography between the two surfaces of the solid polymer which result from the presence of liquid/solid and liquid/air interfaces during the manufacture process. This manufacturing technique presents a potential method for designing scaffold surface topographies using one or more liquid/solid interfaces. However, in order to meet the considerable demands of a structural laryngeal replacement using POSS-PCUU, it is vital that future studies target optimisation of the manufacturing method to improve cellular access to the internal structure. This would provide routes to functional integration with surrounding tissues, including the development of vascular netoworks and extracellular matrices, in turn supporting long-term functionality and internal epithelialization. Although much work is still required, POSS-PCUU has some promising features for the fabrication or coating of advanced bio-integrated implants for unmet healthcare needs.

## Appendix A Supplementary data

Supplementary material Image 1
