# Supplementary material for: Silsesquioxane polymer as a potential scaffold for laryngeal reconstruction
Source: Mater Sci Eng C Mater Biol Appl. 2018 Nov 1;92:565–74. doi: 10.1016/j.msec.2018.07.003 (PMC6134134; doi:10.1016/j.msec.2018.07.003)
Supplement: Supplementary file 1 — Supplementary material [file mmc1.docx]

**SUPPORTING INFORMATION**

**Silsesquioxane Polymer as a Potential Scaffold for Laryngeal Reconstruction.**

**Nazia Mehrban^1¥*^, James Bowen^2¥^, Angela Tait^3^, Arnold Darbyshire^1^, Alex K Virasami^4^, Mark W Lowdell^5^, Martin A Birchall^6*^**

^1^ Division of Surgery, University College London, London, WC1E 6BT, United Kingdom.

^2^ School of Engineering and Innovation, The Open University, Milton Keynes, MK7 6AA, United Kingdom.

^3^ Department of Biochemical Engineering, University College London, London, WC1E 6BT, United Kingdom.

^4^ Department of Histopathology, University College London, London, WC1N 3JH, United Kingdom

^5^Department of Haematology, University College London, London, NW3 2QG, United Kingdom

^6^UCL Ear Institute, University College London, London, WC1X 8DA, United Kingdom.

^¥^These authors contributed equally.

^*^To whom correspondence should be addressed. N. Mehrban (e-mail: [n.mehrban@ucl.ac.uk](mailto:n.mehrban@ucl.ac.uk))


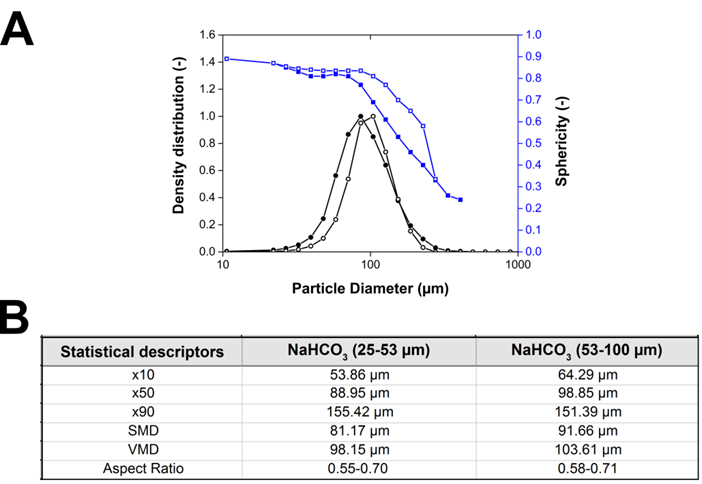


**Figure S1:** NaHCO_3_ size distribution and sphericity. Density distribution of particle diameter and sphericity for (A) 25-53 µm (filled symbol) particles and 53-100 µm (unfilled symbol) particles used for this study with (B) statistical analysis of x10 (the first 10% of the distribution), x50 (the first 50% of the distribution) and x90 (the first 10% of the distribution). SMD refers to the Sauter Mean Diameter and VMD refers to the Volume Mean Diameter.

**
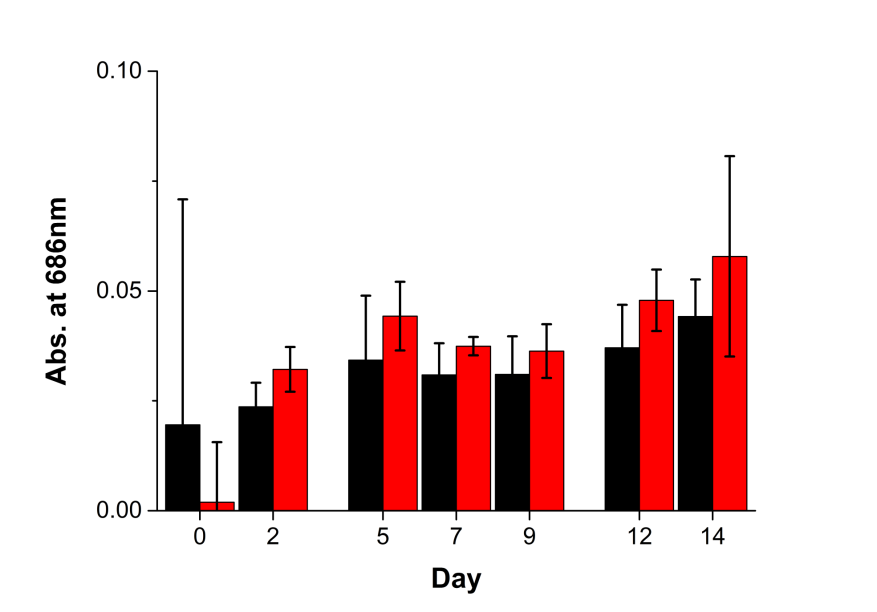
**

**Figure S2:** Bacterial study on polymer type 1 (black) and type 8 (red) over 14 days in tryptic soy broth.


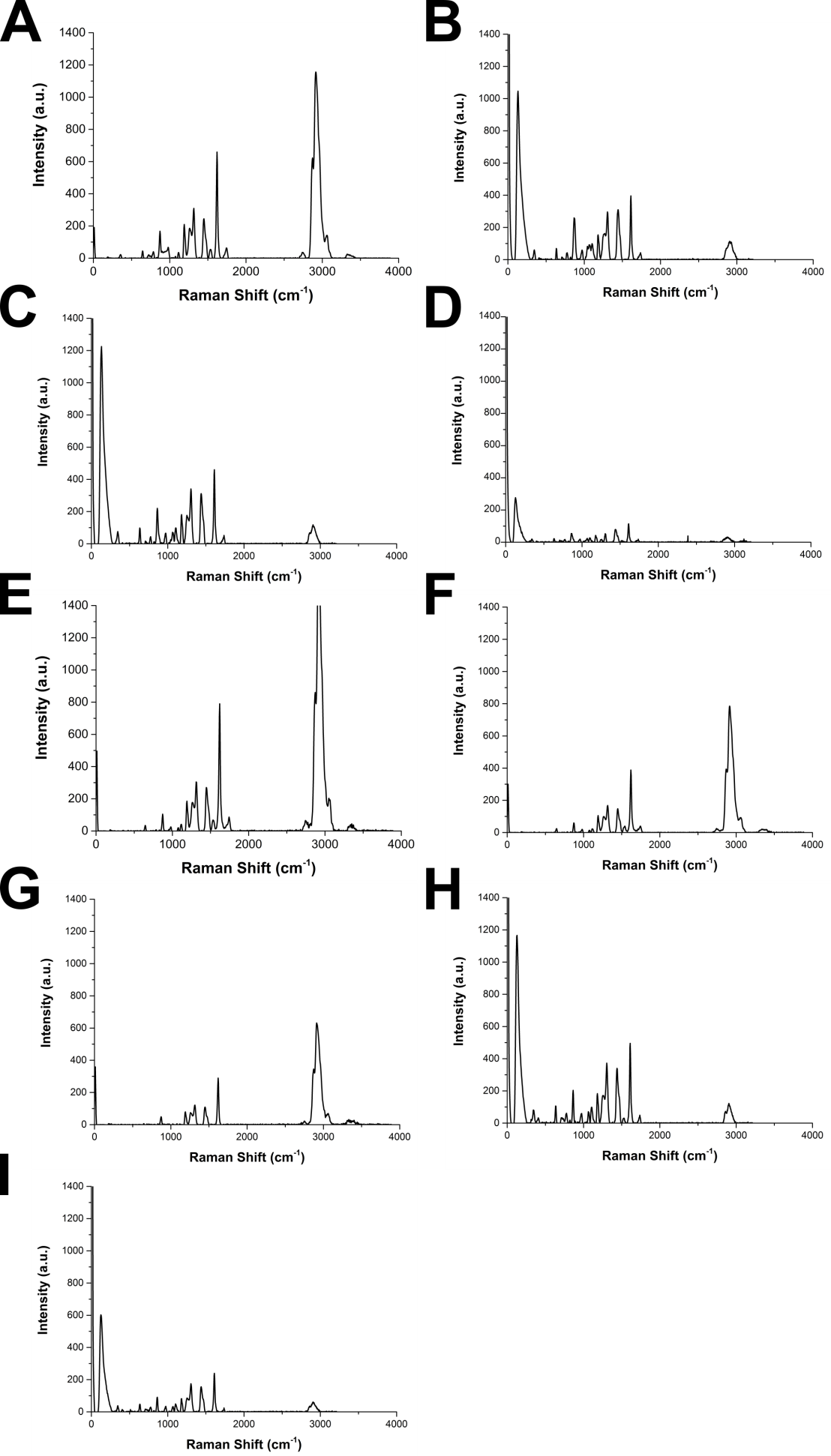


**Figure S3:** Chemical characterisation of POSS-PCUU scaffold surfaces. Representative Raman spectra for (A) polymer type 1, (B) polymer type 2, (C) polymer type 3, (D) polymer type 4, (E) polymer type 5, (F) polymer type 6, (G) polymer type 7, (H) polymer type 8 and (I) polymer type 9. Bond information can be found in Figure 2A.

**
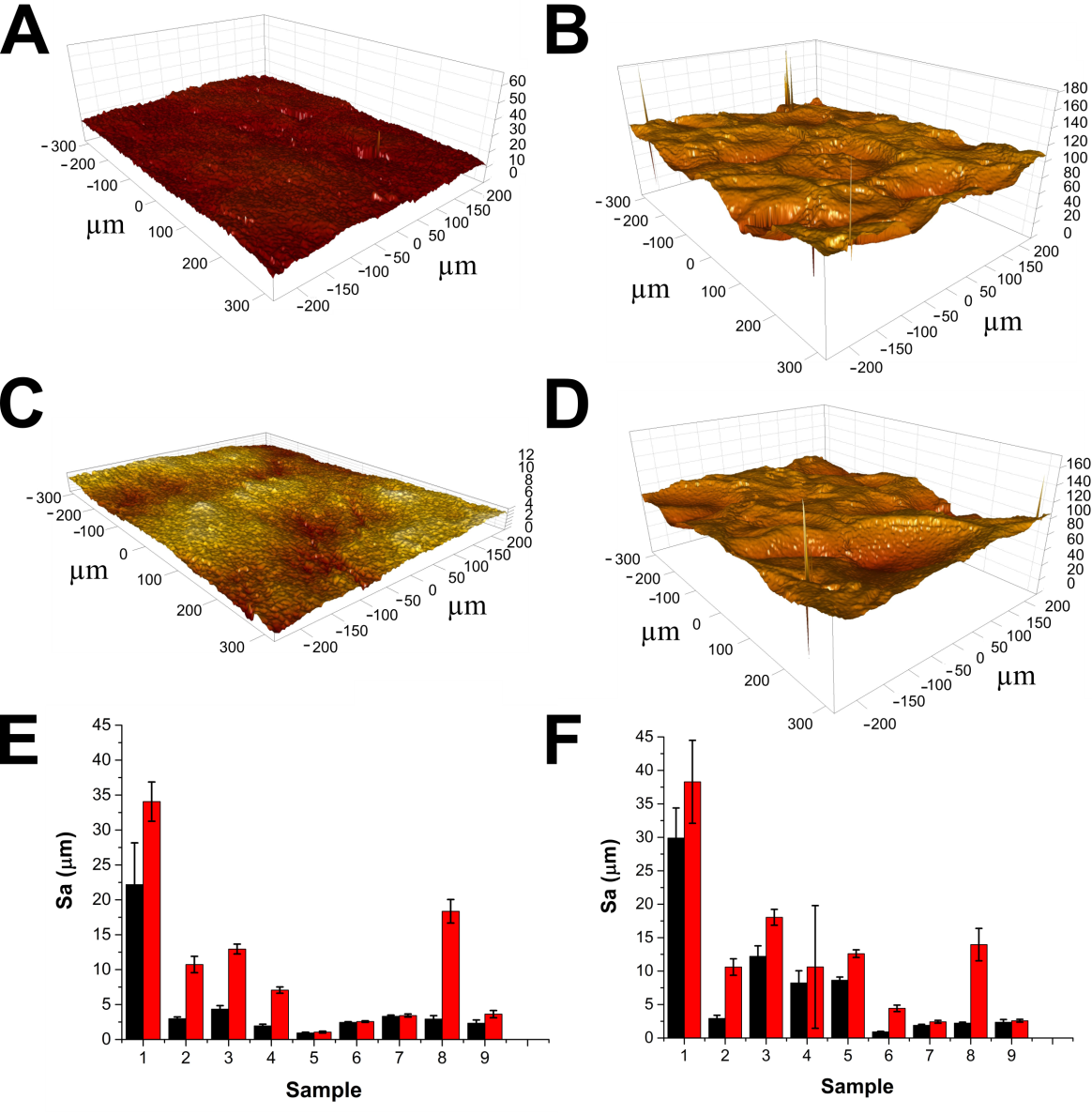
**

**Figure S4:** Polymer surface roughness based on drying methods. Representative 3D profiles of (A) PGI and (B) PAI freeze-dried polymer surfaces and (C) PGI and (D) PAI oven-dried polymer surfaces. Surface roughness has been quantified for (E) freeze-dried and (F) oven-dried polymers of the PGI (black) and PAI (red).

**
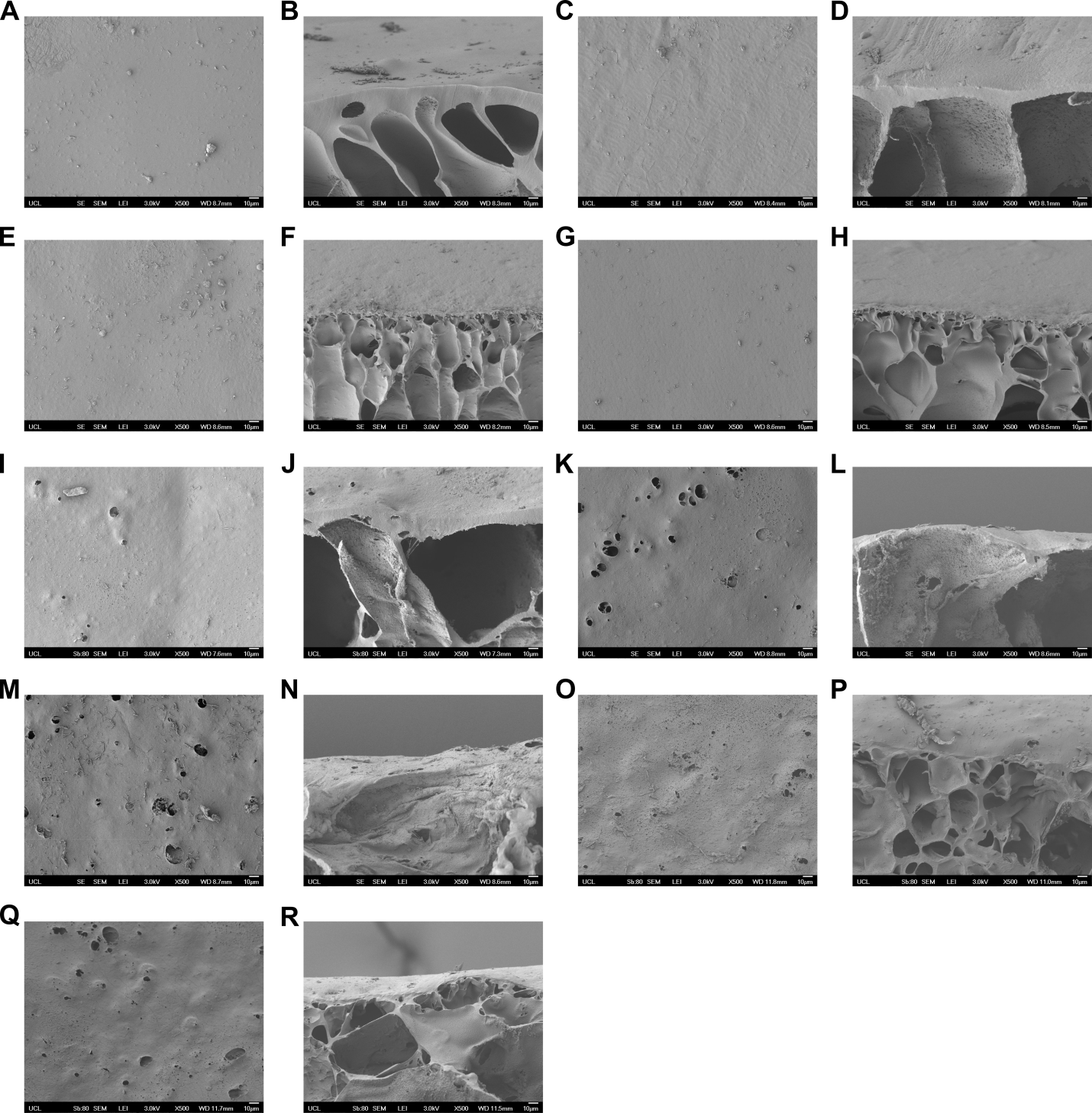
**

**Figure S5:** Surface morphology of POSS-PCUU scaffolds. Scanning electron micrographs of scaffold surface and cross-section for (A, B) polymer type 1, (C, D) polymer type 2, (E, F) polymer type 3, (G, H) polymer type 4, (I, J) polymer type 5, (K, L) polymer type 6, (M, N) polymer type 7, (O, P) polymer type 8 and (Q, R) polymer type 9. Scale bar: 10 µm.

**
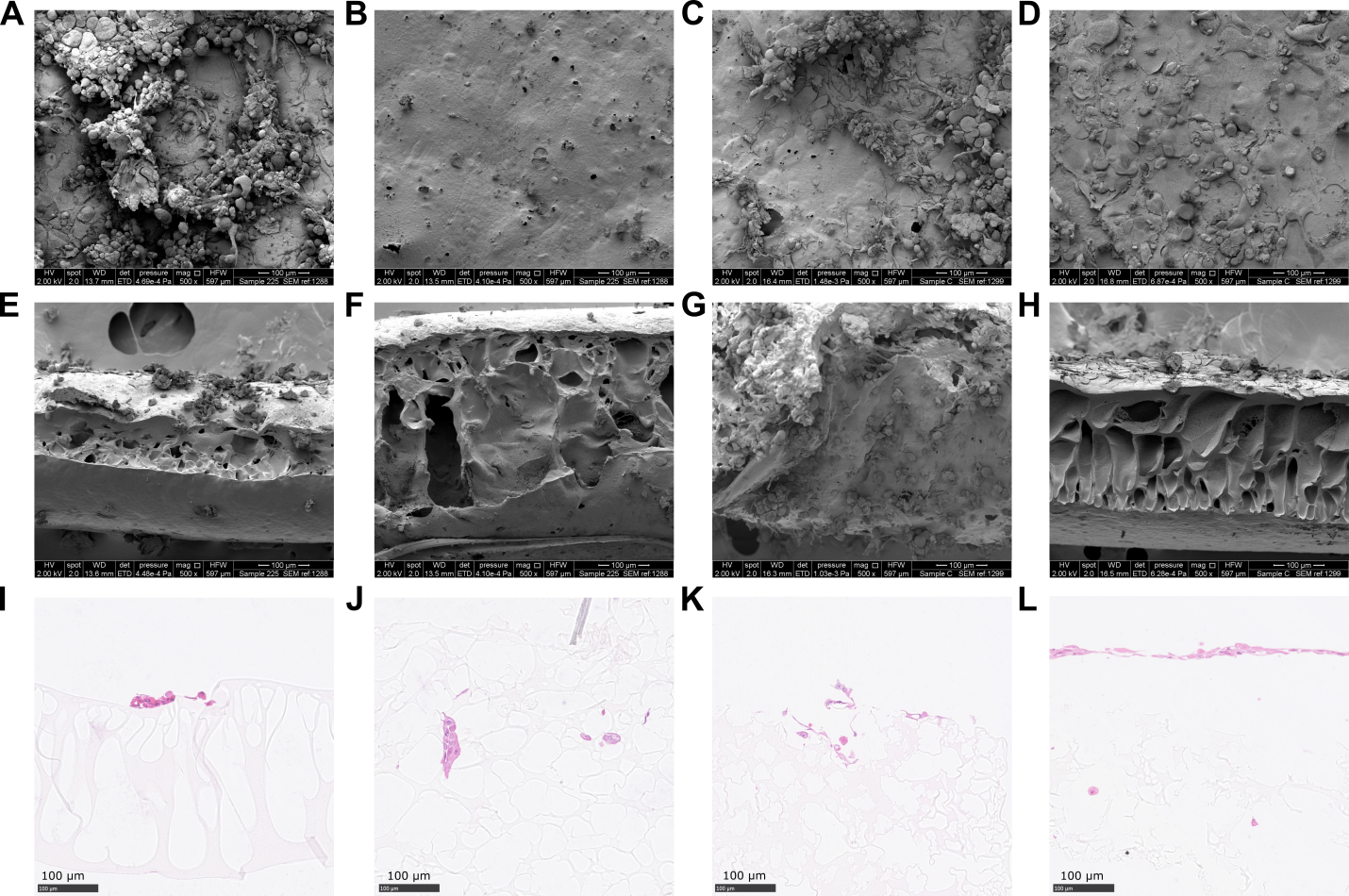
**

**Figure S6:** Cell attachment and migration through POSS-PCUU scaffolds after 1 day in culture. Representative electron micrographs showing cell attachment on the surface of polymer type (A) 1, (B) 5, (C) 8 and (D) 9. Cell migration through POSS-PCUU scaffolds represented by cross-sectional electron micrographs; (E) 1, (F) 5, (G) 8 and (H) 9, haematoxylin-stained (blue-purple) and eosin-stained (pink) cell images; (I) 1, (J) 5, (K) 8 and (L) 9. Scale bar: 100µm.

**MATERIALS AND METHODS**

**Bacterial study** POSS-PCUU samples were autoclaved (121 °C for 20 mins) and incubated in Tryptic Soy Broth (TSB; purchase info) at 20-25°C followed by 30-35°C for 15 days. Aliquots of the TSB were taken at Day 0, 2, 5, 7, 9 and 14 post-incubation and their absorbance measured at 686 nm (Jasco V-630 spectrophotometer, Jasco, UK). Blank TSB solution (containing no polymer) was used as the control and its absorbance subtracted from the final value.
